# Effects of age and sex on eye movement characteristics

**DOI:** 10.1002/npr2.12163

**Published:** 2021-02-21

**Authors:** Junichi Takahashi, Kenichiro Miura, Kentaro Morita, Michiko Fujimoto, Seiko Miyata, Kosuke Okazaki, Junya Matsumoto, Naomi Hasegawa, Yoji Hirano, Hidenaga Yamamori, Yuka Yasuda, Manabu Makinodan, Kiyoto Kasai, Norio Ozaki, Toshiaki Onitsuka, Ryota Hashimoto

**Affiliations:** ^1^ Department of Neuropsychiatry Graduate School of Medical Sciences Kyushu University Fukuoka Japan; ^2^ Department of Pathology of Mental Diseases National Institute of Mental Health National Center of Neurology and Psychiatry Kodaira Japan; ^3^ Department of Rehabilitation University of Tokyo Hospital Tokyo Japan; ^4^ Department of Neuropsychiatry University of Tokyo Tokyo Japan; ^5^ Department of Psychiatry Graduate School of Medicine Osaka University Suita Japan; ^6^ Department of Psychiatry Graduate School of Medicine Nagoya University Nagoya Japan; ^7^ Department of Psychiatry Nara Medical University Kashihara Japan; ^8^ Japan Community Health Care Organization Osaka Hospital Osaka Japan; ^9^ Medical Corporation Foster Osaka Japan; ^10^ The International Research Center for Neurointelligence (WPI‐IRCN) at University of Tokyo Institutes for Advanced Study (UTIAS) Tokyo Japan

**Keywords:** age, eye movement, saccades, sex, smooth pursuit

## Abstract

Abnormal eye movements are often associated with psychiatric disorders. Eye movements are sensorimotor functions of the brain, and aging and sex would affect their characteristics. A precise understanding of normal eye movements is required to distinguish disease‐related abnormalities from natural differences associated with aging or sex. To date, there is no multicohort study examining age‐related dependency and sex effects of eye movements in healthy, normal individuals using large samples to ensure the robustness and reproducibility of the results. In this study, we aimed to provide findings showing the impact of age and sex on eye movement measures. The present study used eye movement measures of more than seven hundred healthy individuals from three large independent cohorts. We herein evaluated eye movement measures quantified by using a set of standard eye movement tests that have been utilized for the examination of patients with schizophrenia. We assessed the statistical significance of the effects of age and sex and its reproducibility across cohorts. We found that 4‐18 out of 35 eye movement measures were significantly correlated with age, depending on the cohort, and that 10 of those, which are related to the fixation and motor control of smooth pursuit and saccades, showed high reproducibility. On the other hand, the effects of sex, if any, were less reproducible. The present results suggest that we should take age into account when we evaluate abnormalities in eye movements.

## Abstract 2

In this study, we aimed to provide findings showing the impact of age and sex on eye movement measures. We found significant effects of age on the eye movement measures related to the fixation and motor control of smooth pursuit and saccades, with high reproducibility. Thus, we should take age into account when we evaluate abnormalities in eye movements.

## Graphical abstract

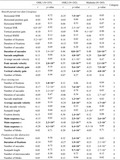

## 1 INTRODUCTION

The current diagnosis of mental illness is based on subjective symptoms, and hence, it often depends on the experience of physicians. To overcome this problem, there is an effort to establish and disseminate guidelines to minimize these variations across Japan^1^. In addition to educational efforts, it is important to establish biological measures that can help support diagnosis/treatment along with such guidelines. Recently, various candidate biomarkers of psychiatric disorders have been proposed^2^, ^3^, ^4^, ^5^, ^6^, ^7^, ^8^, ^9^, ^10^, ^11^, ^12^, ^13^, ^14^, ^15^, and eye movement is one of those with the greatest effect sizes for distinguishing schizophrenia cases from healthy controls^6^.

Eye movement abnormalities in patients with schizophrenia have generally been studied using data obtained for adult subjects without a distinction of sex. However, brain structures are dependent on the age and sex of subjects, and age and sex have often been considered nuisance variables in studying the effects of disease ^16^, ^17^, ^18^, ^19^. Because eye movements are sensorimotor functions of the brain, they might also be dependent on age and sex, as are brain structures. Thus, we sought to determine whether we should consider age and sex as confounding factors when we study eye movement abnormalities. Some previous findings have suggested that aging would affect eye movement characteristics, including lower saccade velocity ^20^, lower visual tracking performance ^21^, ^22^, and poorer antisaccade performance ^23^ in elderly subjects. However, other studies have shown a nonsignificant effect of age on saccade velocity ^24^, ^25^. To date, there has been no multicohort study of the age‐related dependency and sex effects on eye movements in adults using large samples to ensure the robustness and reproducibility of the results.

Overall, a precise understanding of normal eye movements is required to distinguish disease‐related abnormalities from natural differences associated with aging or sex. In this study, we examined the influence of age and sex on eye movement measures in healthy, normal individuals. We evaluated effects on eye movement measures obtained from a set of eye movement tests involving fixation, smooth pursuit, and free‐viewing tasks that were previously utilized for examining abnormalities in schizophrenia ^4^, ^6^. The present study uses three large independent cohorts to evaluate the effects of age and sex and determine their reproducibility among cohorts, and the findings will help in the design of future eye movement studies for not only patients with schizophrenia but also those with other psychiatric disorders and neurological diseases.

## 2 METHODS

### 2.1 Subjects

Three independent cohorts of healthy participants with no history of psychiatric illness and no neurological disorders were involved in this study (Table 1). The first cohort involved 255 subjects recruited at Osaka University where eye movement tests were performed using EyeLink 1000 (OSK1). The second cohort involved 242 subjects recruited at Osaka University in a different time period where eye movement tests were performed using EyeLink 1000 Plus, a newer version of the eye tracker (OSK2). The third cohort (multisite) involved 205 subjects from multiple institutions including the following: Kyushu University (N = 68 (36/32; male/female), age 33.6 ± 8.9 (mean ± SD), [19,59] (range)); University of Tokyo (N = 51 (22/29), age 38.4 ± 8.5, [21,56]); Nagoya University (N = 49 (22/27), age, 39.8 ± 16.6, [19,79]);and Nara Medical University (N = 37 (25/12), age, 25.9 ± 5.2, [16,43]), where EyeLink 1000 Plus was used to perform eye movement tests. All participants provided written informed consent to the study after a full explanation of the procedures. Anonymity was preserved for all participants. The study was performed in accordance with the World Medical Association's Declaration of Helsinki and was approved by the research ethics committees of all the institutions described above and of the National Center of Neurology and Psychiatry.

**TABLE 1:** Demographic information for the subjects

	OSK1 (N = 255)	OSK2 (N = 242)	Multisite (N = 205)
Male/female	145/110	128/114	105/100
Age (mean ± SD)	28.7 ± 11.5	35.0 ± 16.3	34.9 ± 11.7
Age (range)	18‐66	18‐75	16‐79

### 2.2 Eye movement examinations and data collection

The details of the eye movement examinations and data collection have been described elsewhere ^6^, ^26^, ^27^. For cohort OSK1, eye movement examinations consisted of 7 tests (three kinds of fixation tasks with no, near, and far distractor stimuli, three kinds of smooth pursuit tasks involving different stimulus motions including horizontal, slow Lissajous, fast Lissajous, and free‐viewing tasks with 56 images). For cohort OSK2 and the multisite cohort, a smaller battery of eye movement tests was used (fixation task with far distractor stimulus, smooth pursuit task with a fast Lissajous stimulus motion and free‐viewing task with 56 images (OSK2) or 20 images (multisite)). Thirty‐five eye movement measures obtained from the fixation task with a far distractor stimulus, smooth pursuit task with a fast Lissajous stimulus motion, and free‐viewing task were analyzed. The scanpath length measures in the free‐viewing test were given as the median over 56 images for OSK1 and OSK2 and as the mean over 20 images for the multisite cohort.

### 2.3 Statistical analyses

Statistical analyses were performed using Statistical Package for the Social Sciences (SPSS) version 26 (IBM Corp., Armonk, NY). For each of the 35 eye movement measures, the effect of sex was examined using *t* tests, and correlation analyses between age and eye movement measures were performed using Pearson's correlation coefficients. To demonstrate robustness and reproducibility, we categorized the eye movement measures based on the number of cohorts where the *P*‐values were less than 0.05/35 and the largest uncorrected *P*‐value across the three cohorts.

## 3 RESULTS

### 3.1 Effects of age

In cohorts OSK1, OSK2, and the multisite cohort, 4, 18, and 8 out of the 35 eye movement measures, respectively, were significantly correlated with age (see Table 2). We then classified eye movement measures into 4 categories based on the results of statistical analyses. We classified the measure into category 1 when significant correlations were observed in all cohorts, or when significant correlations were observed in two cohorts and the uncorrected *P*‐values across all cohorts were smaller than 0.01. Among those that did not belong to category 1, they were classified into category 2 when there were significant correlations in two cohorts, or when the correlation was significant in one cohort and the uncorrected *P*‐values across all cohorts were smaller than 0.05. The remaining measures were classified into category 3 if the correlation was significant in a cohort; otherwise, they were classified into category 4. According to these criteria, 3 out of the 35 eye movement measures were classified into category 1 (Table 2, bold scripts), 7 into category 2 (Table 2, bold scripts), 9 into category 3, and the remaining 16 into category 4.

**TABLE 2:** Correlation between eye movement measures of age

	OSK1 (N = 255)		OSK2 (N = 242)		Multisite (N = 205)		Category
	*r*	*P*‐value	*r*	*P*‐value	*r*	*P*‐value	
*Smooth pursuit test (fast Lissajous)*							
Horizontal SNR	0.02	0.72	−0.25	**7.5 × 10^−5^**	−0.14	0.05	3
Horizontal position gain	−0.03	0.58	−0.03	0.66	−0.07	0.34	4
Horizontal RMSE	−0.10	0.11	0.04	0.53	0.01	0.87	4
Vertical SNR	6.0 × 10^−3^	0.92	−0.19	2.5 × 10^−3^	−0.13	0.07	4
Vertical position gain	−0.10	0.11	−0.05	0.46	9.1 × 10^−3^	0.90	4
Vertical RMSE	−0.10	0.12	0.09	0.15	0.04	0.53	4
Number of fixations	−5.2 × 10^−3^	0.93	0.14	0.02	0.13	0.07	4
Duration of fixations	0.05	0.41	−0.15	0.02	−0.08	0.24	4
Number of saccades	−0.03	0.69	0.08	0.20	0.15	0.03	4
**Duration of saccades**	0.18	3.4 × 10^−3^	0.46	**8.8 × 10^−14^**	0.48	**3.0 × 10^−13^**	**1**
**Saccade amplitude**	0.13	0.04	0.34	**4.8 × 10^−8^**	0.29	**2.6 × 10^−5^**	**2**
Average saccade velocity	0.12	0.05	0.18	4.1 × 10^−3^	0.05	0.47	4
**Peak saccade velocity**	0.34	**2.0 × 10^−8^**	0.55	**1.0 × 10^−20^**	0.43	**2.1 × 10^−10^**	**1**
**Horizontal velocity gain**	−0.09	0.16	−0.33	**9.6 × 10^−8^**	−0.23	**7.5 × 10^−4^**	**2**
**Vertical velocity gain**	−0.13	0.04	−0.35	**1.4 × 10^−8^**	−0.28	**4.8 × 10^−5^**	**2**
Number of blinks	−0.05	0.44	0.07	0.27	−0.10	0.16	4
*Free‐viewing test*							
Number of fixations	0.23	**1.8 × 10^−4^**	0.12	0.06	0.14	0.04	3
Duration of fixations	−0.17	7.2 × 10^−3^	−0.22	**7.6 × 10^−4^**	−0.12	0.10	3
Number of saccades	0.19	2.2 × 10^−3^	0.02	0.78	0.15	0.03	4
Duration of saccades	−0.05	0.47	−0.06	0.34	0.21	2.7 × 10^−3^	4
Saccade amplitude	−0.11	0.07	−0.21	**1.2 × 10^−3^**	−0.15	0.03	3
**Average saccade velocity**	−0.09	0.14	−0.24	**2.0 × 10^−4^**	−0.26	**1.7 × 10^−4^**	**2**
Peak saccade velocity	0.11	0.09	0.06	0.33	0.06	0.40	4
Scanpath length	0.03	0.68	−0.15	0.02	2.3 × 10^−3^	0.97	4
Fixation density	0.15	0.02	0.30	**1.4 × 10^−6^**	0.12	0.08	3
**Main sequence *v_max_***	−0.15	0.02	−0.23	**2.5 × 10^−4^**	−0.29	**2.6 × 10^−5^**	**2**
**Main sequence *s***	−0.34	**2.3 × 10^−8^**	−0.48	**2.2 × 10^−15^**	−0.36	**1.2 × 10^−7^**	**1**
**Main sequence** *v_0_*	−0.28	**4.1 × 10^−6^**	−0.34	**6.4 × 10^−8^**	−9.3 × 10	0.89	**2**
Number of blinks	0.02	0.71	0.20	**1.4 × 10^−3^**	−0.03	0.71	3
*Fixation test (far distractor)*							
Number of fixations	0.03	0.58	0.32	**2.4 × 10^−7^**	0.15	0.03	3
**Duration of fixations**	−0.14	0.03	−0.27	**1.9 × 10^−5^**	−0.21	2.1 × 10^−3^	**2**
Number of saccades	0.02	0.75	0.29	**4.0 × 10^−6^**	0.21	2.4 × 10^−3^	3
Scanpath length	0.02	0.72	0.31	**9.7 × 10^−7^**	0.21	2.7 × 10^−3^	3
Number of microsaccades	0.07	0.30	0.02	0.73	−0.10	0.15	4
Number of blinks	0.03	0.58	0.13	0.04	−0.16	0.02	4

Note

The names of eye movement tests are written in italics, paradigms within the tests are written in parentheses, and the individual characteristics are written in plain text. The characteristics are written in bold if they were classified as category 1 or 2. Raw *P*‐values are shown. The measures with significant effects of each cohort are shown in Bold script (*P* < 0.05/(35 eye movement scores)=1.4 × 10^−3^).

### 3.2 Effects of sex

No significant effect of sex was found in OSK1 and OSK2, though a significant effect was observed in the multisite cohort (Table 3). Thus, seven eye movement characteristics of smooth pursuit were classified into category 3 based on the same criteria used for the effect of age.

**TABLE 3:** Influence of sex on eye movement measures

	OSK1 (N = 255)		OSK2 (N = 242)		Multisite (N = 205)		Category
	*t‐*value	*P‐*value	*t‐*value	*P‐*value	*t‐*value	*P‐*value	
*Smooth pursuit test (fast Lissajous)*							
Horizontal SNR	−0.21	0.83	0.54	0.59	2.34	0.02	4
Horizontal position gain	−1.15	0.25	−0.47	0.64	0.39	0.70	4
Horizontal RMSE	0.76	0.45	−0.13	0.90	0.19	0.85	4
Vertical SNR	1.59	0.11	2.92	3.9 × 10^−3^	4.75	**3.8 × 10^−6^**	3
Vertical position gain	−1.79	0.07	−1.54	0.12	0.41	0.68	4
Vertical RMSE	−0.93	0.36	−0.90	0.37	−2.36	0.02	4
Number of fixations	1.35	0.18	−0.84	0.40	−1.21	0.23	4
Duration of fixations	−1.58	0.12	0.58	0.57	1.30	0.20	4
Number of saccades	1.56	0.12	0.19	0.85	−1.30	0.19	4
Duration of saccades	−2.12	0.03	−1.23	0.22	−4.99	**1.3 × 10^−6^**	3
Saccade amplitude	−2.54	0.01	−1.50	0.13	−5.16	**5.7 × 10^−7^**	3
Average saccade velocity	−2.80	5.5 × 10^−3^	−1.66	0.10	−3.59	**4.1 × 10^−4^**	3
Peak saccade velocity	−3.20	1.5 × 10^−3^	−1.27	0.20	−5.04	**1.1 × 10^−6^**	3
Horizontal velocity gain	−1.47	0.14	1.86	0.06	3.70	**2.7 × 10^−4^**	3
Vertical velocity gain	0.15	0.88	2.50	0.01	6.13	**4.4 × 10^−9^**	3
Number of blinks	0.07	0.95	−2.95	3.4 × 10^−3^	0.28	0.78	4
*Free‐viewing test*							
Number of fixations	0.64	0.52	0.22	0.83	−0.27	0.78	4
Duration of fixations	−1.03	0.30	−0.69	0.49	−0.99	0.32	4
Number of saccades	0.63	0.53	0.31	0.76	−0.69	0.49	4
Duration of saccades	−0.15	0.88	−0.57	0.57	0.28	0.78	4
Saccade amplitude	−0.20	0.84	0.11	0.92	1.72	0.09	4
Average saccade velocity	−0.18	0.86	0.23	0.82	1.44	0.15	4
Peak saccade velocity	−0.28	0.78	0.05	0.96	0.22	0.83	4
Scanpath length	0.56	0.57	0.59	0.55	1.36	0.17	4
Fixation density	−0.38	0.71	0.55	0.58	−1.08	0.28	4
Main sequence *v_max_*	1.47	0.14	1.81	0.07	0.89	0.37	4
Main sequence *s*	1.42	0.16	1.47	0.14	1.53	0.13	4
Main sequence *v_0_*	0.81	0.42	−0.01	0.99	1.31	0.19	4
Number of blinks	0.33	0.74	−0.44	0.66	0.36	0.72	4
*Fixation test (far distractor)*							
Number of fixations	−0.67	0.50	−0.50	0.62	−1.21	0.23	4
Duration of fixations	−0.06	0.96	0.01	0.99	0.97	0.33	4
Number of saccades	−1.04	0.30	−0.19	0.85	−2.00	0.05	4
Scanpath length	−0.76	0.45	0.78	0.44	−1.05	0.30	4
Number of microsaccades	−0.91	0.37	−2.42	0.02	−0.53	0.60	4
Number of blinks	2.36	0.02	−0.47	0.64	1.32	0.19	4

Note

The names of eye movement tests are written in italics, paradigms within the tests are written in parentheses, and the individual characteristics are written in plain text. Raw *P*‐values are shown. The measures with significant effects of each cohort are shown in Bold script (*P* < 0.05/(35 eye movement scores) = 1.4 × 10^−3^).

## 4 DISCUSSION

In this study, we utilized data from three large cohorts to determine whether age and sex affect eye movement measures obtained from fixation, smooth pursuit, and free‐viewing tasks in healthy participants. The eye movement measures were classified into 4 categories based on their reproducibility of statistical significance across the cohorts: category 1 as “highly reproducible,” category 2 as “reproducible,” category 3 as “less reproducible,” and category 4 as “probably unaffected.” Regarding the effects of age, 10 eye movement characteristics were classified into categories 1 and 2. Although this categorization is rather subjective, it does illustrate an important caveat when dealing with eye movement data for adult participants. We should also note that the correlations were weak (0.1 <= |*r*| < 0.4) for most eye movement measures and moderate (0.4 <= |*r*| < 0.7) for a few measures classified into category 1 ^28^. The effect of sex was significant for seven eye movement characteristics of smooth pursuit only in the multisite cohort. The origin of this discrepancy between the multisite and other cohorts was not identified based on the data. However, regarding the effect of sex, no eye movement measure was classified into category 1 or 2, suggesting low reproducibility and less importance of the effects of sex on eye movement measures.

The eye movement measures that were classified into category 1 or 2 in terms of the effects of age were mostly those related to the motor control of eye movements. In saccades, there is a well‐known relationship called the main sequence; the peak speed increases as the amplitude increases, but it saturates at larger amplitudes ^29^. The maximal speed attainable (main sequence $v_{\max},\;v\left(a\right)=v_{\max}\left(1‐e^{‐s.a}\right)+v_{0}$, where *v*(*a*) denotes peak saccade velocity at amplitude *a*) showed a negative correlation, suggesting that the older the age was, the slower the maximal attainable speed with a change in the overall relationship (main sequence *s* which determines the slope of the peak velocity increase as saccade amplitudes increase (reciprocal of *s* is evaluated) and main sequence *v_0_* which represents the intercept), consistent with a previous finding ^20^. In the free‐viewing test, the effect of age was observed in average saccade velocity but less frequent in saccade amplitude suggesting that, it may also reflect changes in main sequence characteristics. This relationship largely depends on the properties of the brainstem saccade generator and extraocular muscles^30^, suggesting age‐related changes in the low‐level, machine‐like motor control systems of the eyes.

Half of the measures in categories 1 and 2 were those from the smooth pursuit test. There was a marked negative correlation with the horizontal and vertical velocity gains (ie the ratio between the eye velocity and target velocity). This means that the higher the age was, the slower the velocity of smooth tracking relative to target motion. Similar relationships between smooth pursuit and age have been pointed out in previous studies^21^, ^22^. The present result supports their finding using much larger samples obtained from multiple institutes. We also found that the properties of catch‐up saccades were also affected by age, such that larger catch‐up saccades occur in older age (larger saccade amplitude, longer duration of saccades, larger peak saccade velocity), which is thought to be, in part, a secondary effect to compensate for the larger tracking errors resulting from lowered velocity gain. The structures related to smooth pursuit involves several areas of the cerebral cortex, the cerebellum, and the brainstem, as well as the extraocular muscles^31^. Although we could not specify the origins of these age‐related changes, the present results suggest that we need to take age into account when we evaluate smooth pursuit abnormalities.

Various eye movement abnormalities have been reported in patients with schizophrenia^4^, ^32^, ^33^, ^34^, ^35^, ^36^, ^37^, ^38^. Morita et al^6^ developed an integrated eye movement score that distinguished individuals with schizophrenia from healthy controls. The integrated eye movement score consists of three eye movement measures (horizontal position gain in the smooth pursuit test, scanpath length in the free‐viewing test, and duration of fixations in the fixation test). In this study, two of the eye movement measures were not affected by age or sex (horizontal position gain in the smooth pursuit test and scanpath length in the free‐viewing test). However, the duration of fixation in the fixation test showed a correlation with age and was reproducible (category 2; see Table 2), albeit weak, suggesting that fixation is weakly influenced by age, and hence, that Morita's integrated eye movement score may also be influenced, at least in part, by age.

## 5 LIMITATIONS

The findings from this study are limited to those of healthy, normal individuals, and the effects of age and sex in patients were remained unresolved. Further studies are required to clarify age‐ and sex‐related effects on patients with psychiatric/neurological disorders. As the range of age was limited from 16 to 79 in this study, additional studies are necessary to determine the early development of eye movements during childhood and aging in older seniors.

## 6 CONCLUSION

We conclude that not all but some characteristics of fixations, saccades, and smooth pursuit performance depend on age in healthy, normal individuals. Thus, we should take age into account when we evaluate disease‐related abnormalities of eye movements.

## 7 APPROVAL OF THE RESEARCH PROTOCOL BY AN INSTITUTIONAL REVIEW BOARD

The study was performed in accordance with the World Medical Association's Declaration of Helsinki and was approved by the research ethical committees of all the institutions that joined this study (Kyushu Univ, Osaka Univ, Univ Tokyo, Nagoya Univ, Nara Medical Univ) and of the National Center of Neurology and Psychiatry.

## 8 INFORMED CONSENT

All participants provided written consent to the study after a full explanation of the study procedures.

## CONFLICT OF INTEREST

The authors declare no conflicts of interest.

## AUTHOR CONTRIBUTIONS

Junichi Takahashi and Kenichiro Miura were critically involved in the study design, data collection, analysis of the data, interpretation of the data, and writing of the manuscript. Kentaro Morita, Michiko Fujimoto, Seiko Miyata, Kosuke Okazaki, Hidenaga Yamamori, and Yuka Yasuda were involved in the subject recruitment process, and the clinical diagnostic assessments and contributed to the data collection and interpretation. Junya Matsumoto and Naomi Hasegawa were involved in the data analysis. Manabu Makinodan, Kiyoto Kasai, and Norio Ozaki contributed to the interpretation of the data and the writing of the manuscript. Yoji Hirano, Toshiaki Onitsuka, and Ryota Hashimoto supervised the entire project, collected the data, and were critically involved in the study design, interpretation of the data, and the writing of the manuscript. All authors contributed to and approved the final manuscript.

## Data Availability

The data sets generated and/or analyzed during the current study are not publicly available because they contain information that could compromise research participant privacy/consent.
